# Genome-Based Infection Tracking Reveals Dynamics of *Clostridium difficile* Transmission and Disease Recurrence

**DOI:** 10.1093/cid/civ1031

**Published:** 2015-12-18

**Authors:** Nitin Kumar, Fabio Miyajima, Miao He, Paul Roberts, Andrew Swale, Louise Ellison, Derek Pickard, Godfrey Smith, Rebecca Molyneux, Gordon Dougan, Julian Parkhill, Brendan W. Wren, Christopher M. Parry, Munir Pirmohamed, Trevor D. Lawley

**Affiliations:** 1 WellcomeTrust Sanger Institute, Hinxton; 2 University of Liverpool; 3 Royal Liverpool and Broadgreen University Hospitals NHS Trust; 4 London School of Hygiene and Tropical Medicine, United Kingdom

**Keywords:** *C. difficile* 027/ST1, whole-genome sequencing, ward-based transmission, highly contagious individuals, recurrence

## Abstract

***Background.*** Accurate tracking of *Clostridium difficile* transmission within healthcare settings is key to its containment but is hindered by the lack of discriminatory power of standard genotyping methods. We describe a whole-genome phylogenetic-based method to track the transmission of individual clones in infected hospital patients from the epidemic *C. difficile* 027/ST1 lineage, and to distinguish between the 2 causes of recurrent disease, relapse (same strain), or reinfection (different strain).

***Methods.*** We monitored patients with *C. difficile* infection in a UK hospital over a 2-year period. We performed whole-genome sequencing and phylogenetic analysis of 108 strains isolated from symptomatic patients. High-resolution phylogeny was integrated with in-hospital transfers and contact data to create an infection network linking individual patients and specific hospital wards.

***Results.*** Epidemic *C. difficile* 027/ST1 caused the majority of infections during our sampling period. Integration of whole-genome single nucleotide polymorphism (SNP) phylogenetic analysis, which accurately discriminated between 27 distinct SNP genotypes, with patient movement and contact data identified 32 plausible transmission events, including ward-based contamination (66%) or direct donor–recipient contact (34%). Highly contagious donors were identified who contributed to the persistence of clones within distinct hospital wards and the spread of clones between wards, especially in areas of intense turnover. Recurrent cases were identified between 4 and 26 weeks, highlighting the limitation of the standard <8-week cutoff used for patient diagnosis and management.

***Conclusions.*** Genome-based infection tracking to monitor the persistence and spread of *C. difficile* within healthcare facilities could inform infection control and patient management.

## Abstract (precis)

Whole-genome phylogeny and detailed patient movement data identified highly contagious super-spreading donors of *Clostridium difficile* and precisely identified recurrent cases; our approach could guide infection control and patient management to monitor the transmission of *C. difficile* within healthcare facilities.

**(See the Editorial Commentary by Gerding on pages 753–4.)**

*Clostridium difficile* is the most common infectious cause of antibiotic-associated diarrhea in healthcare facilities worldwide [1, 2]. Antibiotic treatment, advanced age, and exposure to a healthcare facility are the major risk factors for *C. difficile* colonization leading to asymptomatic carriage, recurrent diarrhea, pseudomembranous colitis, or death [3, 4].

Unlike other common healthcare-associated pathogens, *C. difficile* produces highly resistant and transmissible spores that confound standard infection control measures [5]. Both asymptomatic carriers and symptomatic patients can excrete spores leading to *C. difficile* spread by direct (person-to-person) or indirect (environmental) modes of transmission. Conventional genotypic methods used for studying *C. difficile* transmission dynamics and epidemiology include polymerase chain reaction (PCR) ribotyping [6], restriction endonuclease analysis (REA) [7], pulsed-field gel electrophoresis [8], toxinotyping [9], multilocus variable-number tandem-repeat analysis [10], and multilocus sequence typing (MLST) [11]. However, these methods are not sufficient to discriminate between genetically monomorphic lineages, such as those from the epidemic *C. difficile* 027/ST1 clade [12].

High-throughput, whole-genome sequencing (WGS) of bacterial pathogens has been successful for investigating *C. difficile* at the global, national, and hospital levels [12–15]. In this study, whole-genome phylogenetic analysis was combined with detailed epidemiological data to monitor *C. difficile* 027/ST1 persistence and transmission within a large university hospital site in Liverpool, United Kingdom, over a 2-year period, revealing novel insight into the dynamics of transmission and recurrent infection.

## METHODS

### Study Population

Patients with *Clostridium difficile* infection (CDI) were recruited from the Royal Liverpool and Broadgreen University Hospitals National Health Service (NHS) Trust, which manages 870 acute beds, between July 2008 and May 2010. Inclusion criteria were adult patients (aged ≥18 years) who developed healthcare-associated diarrhea (ie, passed ≥3 liquid stools in the 24 hours before assessment), had a positive *C. difficile* toxin test (TOX A/B II enzyme-linked immunosorbent assay; Techlab, Blacksburg, Virginia) and a confirmed diagnosis by independent clinicians using national guidelines [16]. A CDI episode was considered nosocomial (healthcare acquired) if the diarrhea arose ≥3 days from the day of hospital admission. Written informed consent was obtained from the relevant patients. The study was approved by the Liverpool Research Ethics Committee (approval reference number 08/H1005/32).

### CDI Screening and Selection of Bacterial Isolates

Only PCR ribotype 027 confirmed isolates were included in this study. Patient-level information collected included data on demographics, patient ward location and movements through the hospital, treatments given, and CDI disease outcomes. The full details of these isolates (including accession numbers) are given in Supplementary Table 1.

### DNA Preparation, Sequencing, Sequence Read Mapping, and SNP Detection

DNA was prepared and sequenced on the Illumina GAIIx platform according to protocols previously described [17]. Paired-end multiplex libraries were created with a 200-bp insertion size. The read length was 76 bp with a minimum coverage of 44-fold. SNPs were identified according to methods previously described [12] using the reference genome of *C. difficile* 027/ST1 strain R20291 [18] (available at https://www.sanger.ac.uk/resources/downloads/bacteria/peptoclostridium-difficile.html).

### Phylogenetic Analysis

Phylogenetic relationships were inferred with the neighbor-joining distance method in SeaView using Jukes–Cantor model [19]. Maximum likelihood analysis was performed using FastTree (–gamma –gtr) [20]. All trees were generated using the Analysis of Phylogenetics and Evolution (APE) package [21].

### Transmission Analysis of *C. difficile* 027/ST1

Transmissions of *C. difficile* 027/ST1 were defined by incorporating phylogenetic information obtained by whole-genome sequencing into a hospital ward–based transmission model [22]. Initially, we identified the potential donor isolate for each test isolate based on its relative position in the phylogenetic tree. For each test isolate, we assumed the donor isolate was of an identical genotype (identical node in the phylogeny) as the test (recipient) isolate, or of an ancestral genotype (ancestral node in the phylogeny) to the test isolate, but was differentiated from the test isolate by ≤2 SNPs [12, 23]. Afterward, based on the hospital ward–based transmission model, epidemiological events were inferred when these pairs shared time on the same ward, either (1) after the donor's sample, or before the recipient's sample, or (2) before both pair's samples were taken. For every successful transmission, the “minimum infectious period” was the required criteria and was defined as the time between the first sample from the potential donor and ward contact with the recipient, whereas the time between this ward contact and the recipient's first sample was termed the “incubation period” (Supplementary Figure 3). Maximum infectious periods of 8 weeks, incubation periods of 12 weeks, and ward contamination time (time from donor discharge to recipient admission) of 26 weeks were allowed. Four types of ward-based contact (2 “directional,” 1 “nondirectional,” and 1 “ward contamination”) were assessed to identify potential transmission links [22].

### Statistical Analysis

Analysis of epidemiological and medicinal data associated with potential donors was performed using GraphPad Prism 6 software by unpaired 2-tailed *t* test. *P* values <.05 were considered significant.

## RESULTS

### CDI During the Sampling Period

**Figure 1.**
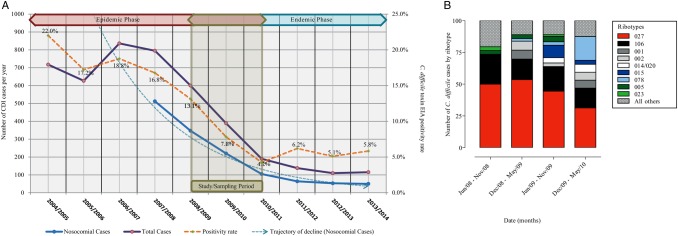
Trends of *Clostridium difficile* incidence in the Royal Liverpool and Broadgreen University Hospitals National Health Service Trust complex. *A*, Incidences of *C. difficile* cases during the period from 2004 to 2013. Sampling period was between July 2008 and May 2010. The right y-axis is absolute number of *C. difficile* infection (CDI) cases and the left y-axis is percentage of positive *C. difficile* toxin enzyme immunoassays (EIAs). Different CDI cases are shown in straight blue (nosocomial) and straight purple (total) lines. Trajectory of decline in nosocomial cases is shown by blue broken line. Percentage of positive *C. difficile* toxin EIA is shown by gold broken line. *B*, Percentage of CDI cases based on most frequent *C. difficile* polymerase chain reaction ribotypes is shown by red (027/ST1), black (106), dark gray (001), gray (002), white (014/020), dark blue (015), sky blue (078), dark green (005), light green (023), and black dotted (other ribotypes) boxes during sampling period.

More than 10 580 hospital and community samples from 7048 patients were screened and 801 were confirmed as CDI, a positivity rate of 7.6%. Of these, 616 samples originated from hospitalized patients and consisted of 453 nosocomial (healthcare acquired) and 163 community-associated CDI cases (Figure 1*A*). From the retrievable samples cultured (463/616), 446 isolates were obtained (96% recovery rate) and were subjected to PCR ribotyping (Figure 1*A*).

In response to enhanced infection control measures that were gradually introduced prior to and throughout the sampling period, monthly CDI figures gradually dropped from 38 in July 2008 (annual total figure of 600, of which 353 were nosocomial) to 16 in May 2010 (annual total of 190, of which 105 were nosocomial) (Figure 1*A*). The control measures (Supplementary Table 2) included more stringent antibiotic prescription policy, improved disinfection of wards, opening of a dedicated isolation ward, and more effective testing and management of patients. This was also consistent with UK-wide data during a comparable period [22].

During the entire sampling period, *C. difficile* 027/ST1 was the most prevalent PCR ribotype, ranging from 50% in 2008 to 31% in 2010 (Figure 1*B*). Of these *C. difficile* 027/ST1 samples, 108 (34%) were sampled and sequenced from 87 patients with confirmed *C. difficile* infection (Supplementary Figure 1), including multiple samples from 14 patients with recurrent infection.

### Genome-Based Infection Tracking of *C. difficile*

To distinguish between strains from the genetically monomorphic 027/ST1 lineage [12], we performed whole-genome SNP discovery of the *C. difficile* 027/ST1 sample collection and constructed a high-resolution phylogenetic tree based on SNPs from the 3.8-Mb nonrepetitive core genome (95% of the genome). The SNP-based phylogeny subdivided the 108 sequenced isolates from the single MLST genotype into 27 distinct SNP genotypes (grouping of strains based on identical SNP patterns) that were differentiated by ≤70 SNPs, vastly improving the discriminatory power over MLST genotyping (Supplementary Figure 2).

To explore the dynamics of *C. difficile* 027/ST1 persistence and spread among patients, we adopted an epidemiological model [22] that identifies the most plausible *C. difficile* transmission events based on shared time and wards of patients and *C. difficile* MLST genotype. This model distinguishes between types of transmission events (Supplementary Figure 3) including directional (transmission occurs on a shared ward after donor's and before recipient's positive samples), nondirectional (transmission on a shared ward preceding collection of positive samples from both the donor and recipient), and ward (transmission on a shared ward after donor's discharge—no temporal overlap between donor and recipient) contamination. In addition, we increased the resolution of the model by incorporating all recorded patient movements between wards to track the spread of *C. difficile* 027 within the hospital.

**Figure 2.**
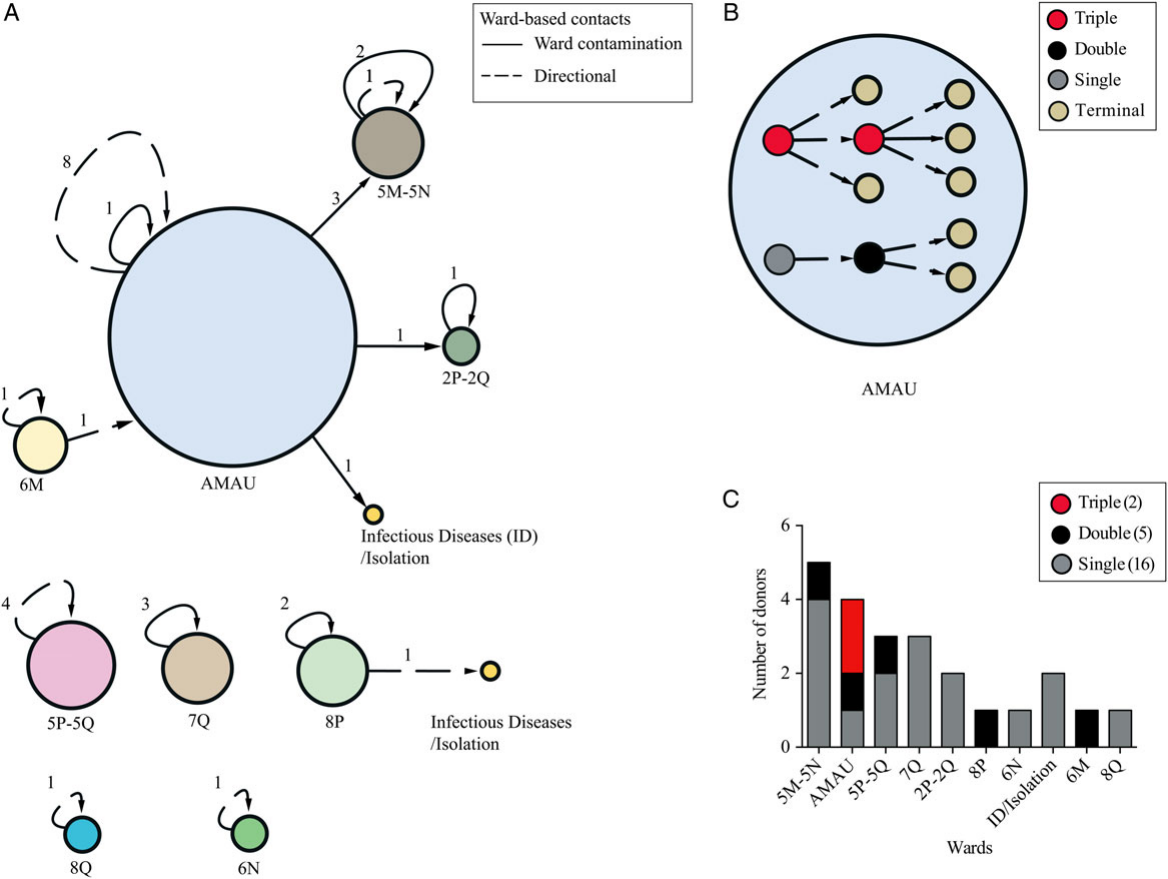
Hospital ward–level transmission analysis based on single-nucleotide polymorphism (SNP) genotypes of 027/ST1 isolates. *A*, Distribution of ward-based transmissions in different wards/units. Node sizes are proportional to the number of transmission events. Different types of ward-based contacts are shown in broken lines (directional) and straight lines (ward contamination). Node labels indicate floor number and specialty ward. Acute medical assessment unit (AMAU) is located at ground floor. Edge labels indicate number of events. *B*, Transmission network between sampled patients within the AMAU. Different types of donors are shown by red (triple), gray (double), black (single), and khaki (terminal). Line code for different types of ward-based contacts is the same as for (*A*). *C*, Bar plot showing the distribution of different type of donors within specific wards (floor number and specialty ward). Color-coding for different types of donors is the same as for (*B*).

By combining this high-resolution SNP-based phylogeny with our spatial–temporal model, we were able to identify 32 transmission events, including 21 ward-based transmission events and 11 directional transmission events, both within and between specific wards. We identified a transmission network that links 11 wards around the hospital, including the acute medicine assessment unit (AMAU) at the ground floor and then specialty wards linked with admissions associated with healthcare of elderly persons, gastrointestinal, renal/dialysis, hematology, and surgical procedures. These could be geographically mapped to the following areas: 2P–2Q (second floor), 5M–5N and 5P–5Q (fifth floor), 6M–6N (sixth floor), 7Q (seventh floor), and 8P–8Q (eighth floor), as well as to the infectious diseases/isolation wards on the third floor that were utilized as containment of acute confirmed cases (Figure 2*A*). Using the transmission network (Figure 2*A*), we identified that the majority of transmission events occurred within specific wards (25/32 [78%]), most commonly within the AMAU (9 transmission events). In contrast, we did not observe any transmission events that occurred within the infectious diseases/isolation wards, where stringent infection control practices were in place.

We found that 22% of the transmission events spread *C. difficile* 027/ST1 between wards. The majority of transmission events that occurred between wards originated from the AMAU and circulated to specialty wards 5M–5N (3 transmission events), 2P–2Q (1 transmission event), and the infectious diseases/isolation wards on the third floor (1 transmission event). We did not observe any transmission events between the wards 5P–5Q, 6N, 7Q, or 8Q (Figure 2*A*). This method allowed us to establish a highly discriminatory method (Supplementary Figure 4) to track the spread of individual *C. difficile* 027 clones within a hospital setting.

### Identification of Highly Contagious Donors

The ward-based network analysis (Figure 2*A*) highlighted high levels of transmission within the AMAU, and so we next investigated the spread of *C. difficile* 027/ST1 within this ward. We constructed a detailed patient-to-patient transmission network of 11 infected patients (Figure 2*B*) that are linked by 9 transmission events: 8 ward-based and 1 directional. This patient-to-patient transmission network identified donors who had transmitted to multiple recipients, suggesting the presence of highly contagious individuals. Among the 4 donors in the AMAU, 1 donor infected a single recipient, another donor infected 2 recipients, and 2 donors each infected 3 recipients. We could not identify triple donors in any other wards, but we did identify 4 double donors in the 8P, 5P–5Q, 6M, and 5M–5N wards. A total of 15 single donors were identified in the 6N, 2P–2Q, 5P–5Q, 7Q, 5M–5N, 8Q, and infectious diseases/isolation wards (Figure 2*C*). Thus, we identified that the AMAU contained the most contagious individuals.

Next, we were interested in identifying any features associated with donor transmission frequency. Based on the patient movement and contact data, we calculated both the incubation period and infectious period for each transmission event (Supplementary Figure 3). We found that the donor incubation period was not associated (*P* > .05) with the frequency of transmission (Supplementary Figure 5), but the donor infectious period was significantly shorter for triple donors compared to that of single donors (*P* < .05; Supplementary Figure 5*A*).

We also investigated if higher-frequency transmission can be linked to patient treatments or in-hospital transfers. We did not find any significant association between this and the length of stay in hospital nor the number of patient movements between wards (Supplementary Figure 5). Furthermore, there was no association (*P* > .05) with patient treatments including the duration or number of antibiotic treatment, and exposure to proton pump inhibitors (Supplementary Figure 6). These results suggest that the only observable feature associated with increased transmission from donors was a short infectious period, implying a brief but highly contagious state.

### Identification of Source of Recurrent CDI

**Figure 3.**
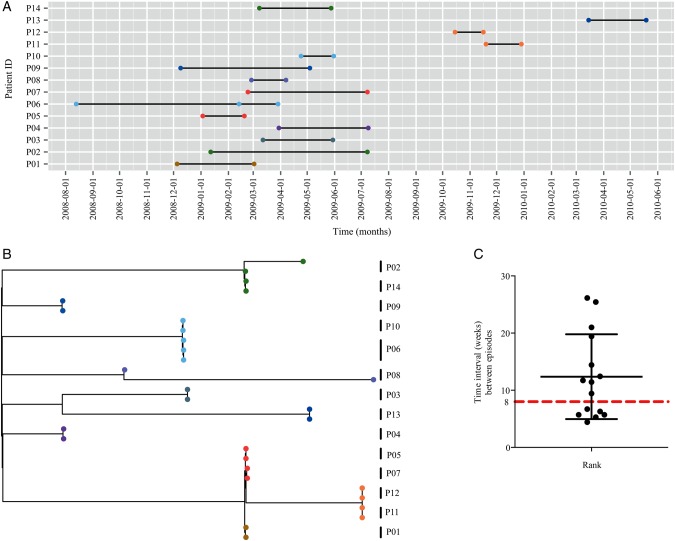
Classification of recurrent disease as relapse or reinfection based on single-nucleotide polymorphism (SNP) genotypes. *A*, Temporal graph of 29 isolates (circle) of 027/ST1 isolated from 14 patients (P1–P14). The isolates are colored on the basis of their SNP genotype group as in Supplementary Figure 2. *B*, Maximum likelihood tree based on SNP differences across the core genome showing the position of 29 recurrent isolates of 027/ST1 isolated from 14 patients (P01–P14). Strain nodes are colored on the basis of their SNP genotype group as in Supplementary Figure 2. *C*, Recurrent episodes isolated from 14 patients (P01–P14) with a range of 4 and 26 weeks. Abbreviation: ID, identification.

The currently used clinical definition of CDI defines subsequent infection as a recurrent infection if the second episode occurs within 8 weeks of the initial episode, or a new infection if the second episode occurs after 8 weeks [24]. Recurrent CDI may result from either relapse with the same strain or reinfection with a different strain [3]. This distinction between relapse and reinfection is not possible in clinical practice, but is an important distinction to make, as identifying the source of the recurrent infection could guide patient management policies. Our sampling included isolates from 14 patients sampled at each disease episode (Figure 3*A*).

We sequenced isolates from these 14 multiepisode patients (P1–P14) (Figure 3*A*) and performed whole-genome SNP discovery of 29 sequenced isolates to construct a high-resolution maximum likelihood phylogeny. The maximum likelihood tree (Figure 3*B*) revealed that the paired episodes in 12 of the 14 patients shared the exact SNP genotype (identical genome). However, we observed that paired isolates from patient 2 (P02 in Figure 3) differed by 1 SNP, and paired isolates from patient 8 (P08 in Figure 3) differed by 4 SNPs. This analysis indicates that the main source of recurrent *C. difficile* 027 disease was the original infecting strain; we are thus confident that 12 of the patients had relapsing infection.

Interestingly, in contrast to the 6 recurrent infections and 8 new infections in our sampling based on the existing clinical definition of recurrent CDI (Figure 3*A*), we noted that the time between the initial episode and the second episode was well beyond the 8-week clinical criteria. On average, relapsing disease occurred around 12 weeks after the initial episode, with a range of 4–26 weeks (Figure 3*C*). These results suggest that the current clinical definition of recurrent CDI may misclassify the majority of recurrent cases in surveillance data.

## DISCUSSION

Here we present a novel application of whole-genome phylogenetics to track the movements of individual *C. difficile* 027/ST1 clones persisting within and transmitting between patients that cannot be accomplished with standard genotyping methods [22] or stringent SNP cutoffs based on whole-genome sequencing [23]. The power of our approach is highlighted by the fact that we could discriminate 108 strains from the *C. difficile* 027/ST1 genotype into 27 distinct SNP genotypes and delineate their precise evolutionary relationships. Our method to investigate transmissions suggests that the majority of *C. difficile* 027/ST1 strains at our hospital were circulated by ward-based contamination (60%). In contrast, previous studies of transmissions [22, 23] using different approaches suggested low rates of *C. difficile* transmission by ward-based contamination in different hospitals. We believe each hospital maintains different rates and types of *C. difficile* transmissions linked to infection control practices and types of circulating *C. difficile* variants, and therefore one general view of *C. difficile* epidemiology and infection dynamics cannot encompass every hospital.

Whole-genome sequencing has provided a unique benefit to infection control management by identifying “super-spreaders” who can infect a disproportionately high number of susceptible individuals [25–27]. We identified highly contagious donors of *C. difficile* who were potential super-spreaders within the AMAU responsible for spreading infection throughout the hospital. The identification of conditions that facilitate an increase in frequency of transmission is a priority during endemic and epidemic situations to control or eliminate infections. The only notable feature of theses highly contagious donors was the relatively short infectious period, suggesting a brief but highly contagious state. The AMAU is characterized by a high patient turnover rate, high patient contact rates, and difficulties in carrying out disinfection at the same stringency as the other wards. Thus, infection control measures should be focused on these areas.

Another application of SNP genotyping includes the study of persistent infection in individual patients. In particular, it is not possible to distinguish between relapse (same strain) and reinfection (different strain) when the same ribotype is involved using standard genotyping methods. This is an important distinction to make as a relapse would signify partial cure or incomplete eradication of the organism, whereas reinfection would signify an individual with a much higher propensity of developing CDI. Therefore, establishing the precise form of recurrent infection could effectively guide patient management and treatment policies. Our results based on SNP phylogeny indicate that the definition of recurrent CDI may need to be extended beyond the 8-week clinical criteria, as we have found that high rates of relapse (same strain) frequently occur beyond the 8-week clinical criteria, and highlight that *C. difficile* 027/ST1 recurrence can occur as long as 26 weeks after the initial infection. Our claims are further supported by a growing body of evidence that eradication of *C. difficile* with treatment occurs only in a minority of treated patients [28, 29] and that stool cultures post-CDI may remain positive for >26 weeks in some patients [29]. Moreover, detailed study of recurrent cases, including a larger sample size and multiple infectious periods, would aid in identifying the factors associated with recurrent disease and demonstrate the efficacy of recurrence classification based on SNP genotypes.

Our study has some limitations. First, our study data have a risk of selection bias as we were not able to include a proportion of *C. difficile* 027/ST1 isolates due to logistic and technical limitations associated with the lack of clinical, epidemiological, and movement data for certain cases, as well as the fact that genomes of certain isolates did not pass quality control measures due to the insufficient levels of genome coverage. Second, although we identified robust transmission networks with only one-third of the *C. difficile* 027/ST1 strains sequenced, our infectious network analysis may have missed transmission events that occurred between symptomatic patients or asymptomatic carriers. Even so, the analysis was able to monitor *C. difficile* spread at an unprecedented resolution that allowed us to identify highly contagious individuals or super-spreaders. We have not explored any clinical features of highly contagious individuals, and this warrants further investigation. Second, our recurrent analysis may have misclassified the episodes of patients 2 and 8, as these patients could have acquired isolates of the same genotypes (point mutation) from exogenous sources after the initial infection. However, our findings emphasize the importance of accurate identification of recurrent cases that would facilitate patient management to detect précised rates of disease of the hospital, which would impact on economic rates of the hospital. Recently, strict single-nucleotide variant cutoff criterion [30] was suggested to classify recurrent cases, but this study did not infer the evolutionary relationship between strains, which we show is essential to discriminate 2 causes of recurrent disease.

In conclusion, we monitored the persistence and spread of the epidemic *C. difficile* 027/ST1 lineage between symptomatic hospital patients who could be the major source of CDI transmissions [28, 31]. Phylogenetic SNP genotyping was able to detect precise patient-to-patient transmissions and recurrent events of CDI. We envisage that genome databases with relevant metadata will serve as a common, open-access resource that can be exploited to identify and track *C. difficile* within their local region through whole-genome sequencing or other comparative measures such as SNP typing.

## Supplementary Data

Supplementary materials are available at http://cid.oxfordjournals.org. Consisting of data provided by the author to benefit the reader, the posted materials are not copyedited and are the sole responsibility of the author, so questions or comments should be addressed to the author.

Supplementary Data
