# Supplementary material for: Genome-Based Infection Tracking Reveals Dynamics of Clostridium difficile Transmission and Disease Recurrence
Source: Clin Infect Dis. 2015 Dec 18;62(6):746–52. doi: 10.1093/cid/civ1031 (PMC4772841; doi:10.1093/cid/civ1031)
Supplement: Supplementary Data [file supp_62_6_746__index.html]

Genome-based infection tracking reveals dynamics of Clostridium difficile transmission and disease recurrence — Genome-Based Infection Tracking Reveals Dynamics of Clostridium difficile Transmission and Disease Recurrence — Genome-Based Infection Tracking Reveals Dynamics of Clostridium difficile Transmission and Disease Recurrence — Supplementary Data 

# Genome-Based Infection Tracking Reveals Dynamics of *Clostridium difficile* Transmission and Disease Recurrence

## Supplementary Data

Supplementary Data

- Supplementary Data - Docx file
