# Supplementary material for: Genome-Based Infection Tracking Reveals Dynamics of Clostridium difficile Transmission and Disease Recurrence
Source: Clin Infect Dis. 2015 Dec 18;62(6):746–52. doi: 10.1093/cid/civ1031 (PMC4772841; doi:10.1093/cid/civ1031)
Supplement: Supplementary Data [file supp_civ1031_civ1031supp.docx]

**Supplementary information for:**

**Genome-based infection tracking reveals dynamics of *Clostridium difficile* transmission super-spreading and disease recurrence**

Nitin Kumar^1§^, Fabio Miyajima^2§^, Miao He^1^, Paul Roberts^3^, Andrew Swale^2^, Louise Ellison^1^, Derek Pickard^1^, Godfrey Smith^3^, Rebecca Molyneux^3^, Gordon Dougan^1^, Julian Parkhill^1^, Brendan W. Wren^4^, Christopher M. Parry^4^, Munir Pirmohamed^2^, Trevor D. Lawley^1*^

^*^ [tl2@sanger.ac.uk](mailto:tl2@sanger.ac.uk)

**Supplementary Table 1 – List of *Clostridium difficile* 027 strains used in this study, the Royal Liverpool and Broadgreen University Hospitals NHS Trust, July 2008 - May 2010**

| **Strain** | **R_L#T^a^** | **ENA ID^b^** |
| --- | --- | --- |
| Liv001^c^ | 4350_8#1 | ERS124182^d^ |
| Liv002^c^ | 4350_8#11 | ERS124183 |
| Liv003^c^ | 4350_8#8 | ERS124186 |
| Liv004^c^ | 4350_8#7 | ERS124187 |
| Liv005^c^ | 4350_8#6 | ERS124188 |
| Liv006^c^ | 5673_7#11 | ERS017273 |
| Liv007^c^ | 4350_8#4 | ERS124190 |
| Liv008^c^ | 5673_6#8 | ERS017223 |
| Liv009^c^ | 5673_6#9 | ERS017224 |
| Liv010^c^ | 5673_7#12 | ERS017274 |
| Liv011^c^ | 4350_3#1 | ERS124194 |
| Liv012^c^ | 4350_3#11 | ERS124195 |
| Liv013^c^ | 4350_3#8 | ERS124198 |
| Liv014^c^ | 4350_3#7 | ERS124199 |
| Liv015^c^ | 4350_3#6 | ERS124200 |
| Liv016^c^ | 5589_7#10 | ERS017213 |
| Liv017^c^ | 5589_7#11 | ERS017214 |
| Liv018^c^ | 5589_7#12 | ERS017215 |
| Liv019^c^ | 5673_6#1 | ERS017216 |
| Liv020^c^ | 5673_6#2 | ERS017217 |
| Liv021^c^ | 5673_6#3 | ERS017218 |
| Liv038 | 5295_3#2 | ERS012866 |
| Liv040^c^ | 5295_3#4 | ERS012868 |
| Liv041^c^ | 5295_3#5 | ERS012869 |
| Liv048^c^ | 5295_3#8 | ERS012872 |
| Liv049^c^ | 5295_3#9 | ERS012873 |
| Liv051 | 5295_3#11 | ERS012875 |
| Liv052 | 5295_3#12 | ERS012876 |
| Liv053 | 5295_5#1 | ERS012877 |
| Liv054 | 5295_5#2 | ERS012878 |
| Liv055 | 5295_5#3 | ERS012879 |
| Liv056 | 5295_5#4 | ERS012880 |
| Liv057 | 5295_5#5 | ERS012881 |
| Liv058 | 5295_5#6 | ERS012882 |
| Liv059 | 5295_5#7 | ERS012883^d^ |
| Liv060 | 5295_5#8 | ERS012884 |
| Liv061 | 5295_5#9 | ERS012885 |
| Liv062 | 5295_5#10 | ERS012886 |
| Liv063 | 5295_5#11 | ERS012887 |
| Liv064 | 5295_5#12 | ERS012888 |
| Liv065 | 5408_1#1 | ERS012889 |
| Liv066 | 5408_1#2 | ERS012890 |
| Liv067 | 5408_1#3 | ERS012891 |
| Liv068 | 5408_1#4 | ERS012892 |
| Liv069 | 5408_1#5 | ERS012893 |
| Liv070 | 5408_1#6 | ERS012894 |
| Liv071 | 5408_1#7 | ERS012895 |
| Liv072 | 5408_1#8 | ERS012896 |
| Liv073 | 5408_1#9 | ERS012897 |
| Liv074 | 5408_1#10 | ERS012898 |
| Liv075 | 5408_1#12 | ERS012900 |
| Liv076 | 5295_6#1 | ERS012901 |
| Liv077 | 5295_6#2 | ERS012902 |
| Liv078 | 5295_6#3 | ERS012903 |
| Liv079 | 5295_6#4 | ERS012904 |
| Liv081 | 5295_6#6 | ERS012906 |
| Liv082 | 5295_6#7 | ERS012907 |
| Liv083 | 5295_6#8 | ERS012908 |
| Liv084 | 5295_6#9 | ERS012909 |
| Liv085 | 5295_6#10 | ERS012910 |
| Liv086 | 5295_6#11 | ERS012911 |
| Liv087 | 5295_6#12 | ERS012912 |
| Liv088 | 5295_7#1 | ERS012913 |
| Liv089 | 5295_7#2 | ERS012914 |
| Liv090 | 5295_7#3 | ERS012915 |
| Liv091 | 5295_7#4 | ERS012916 |
| Liv092 | 5295_7#5 | ERS012917 |
| Liv093 | 5295_7#6 | ERS012918 |
| Liv094 | 5295_7#7 | ERS012919 |
| Liv095 | 5295_7#8 | ERS012920 |
| Liv096 | 5295_7#10 | ERS012922 |
| Liv097 | 5295_7#11 | ERS012923 |
| Liv098 | 5295_7#12 | ERS012924 |
| Liv100 | 5295_8#2 | ERS012926^d^ |
| Liv101 | 5295_8#3 | ERS012927 |
| Liv102 | 5295_8#4 | ERS012928 |
| Liv103 | 5295_8#5 | ERS012929 |
| Liv104^c^ | 5295_8#6 | ERS012930 |
| Liv127^c^ | 5295_8#7 | ERS012931 |
| Liv131 | 5295_8#8 | ERS012932 |
| Liv132 | 5295_8#9 | ERS012933 |
| Liv136 | 5295_8#10 | ERS012934 |
| Liv145^c^ | 5789_3#3 | ERS017156 |
| Liv146^c^ | 5578_7#2 | ERS017157 |
| Liv147 | 5578_7#3 | ERS017158 |
| Liv148 | 5578_7#4 | ERS017159 |
| Liv149 | 5578_7#5 | ERS017160 |
| Liv170 | 5578_3#4 | ERS017183 |
| Liv171 | 5578_3#5 | ERS017184 |
| Liv172 | 5789_3#8 | ERS017185 |
| Liv173 | 5578_3#7 | ERS017186 |
| Liv174 | 5578_3#9 | ERS017188 |
| Liv175 | 5578_3#10 | ERS017189 |
| Liv176 | 5578_3#11 | ERS017190 |
| Liv177 | 5578_3#12 | ERS017191 |
| Liv178 | 5589_8#1 | ERS017192 |
| Liv179 | 5589_8#2 | ERS017193 |
| Liv180 | 5589_8#3 | ERS017194^d^ |
| Liv181 | 5589_8#4 | ERS017195 |
| Liv182 | 5589_8#5 | ERS017196 |
| Liv183 | 5589_8#6 | ERS017197 |
| Liv184 | 5589_8#7 | ERS017198 |
| Liv185 | 5589_8#9 | ERS017200 |
| Liv186 | 5589_8#10 | ERS017201 |
| Liv187 | 5589_8#11 | ERS017202 |
| Liv188 | 5589_8#12 | ERS017203^d^ |
| Liv189 | 5589_7#1 | ERS017204 |
| Liv190 | 5589_7#2 | ERS017205 |

a: run, lane and tag numbers

b: European Nucleotide Archive ID

c: Multiple episodes

d: He and colleagues [[1](#_ENREF_1)]

**Supplementary Table 2 – Major interventional measures introduced at RLUBHT between July 2008 - May 2010**

| **Date** | **Major Intervention Procedure** |
| --- | --- |
| April 2007 | Enhanced surveillance of *Clostridium difficile* Infection and notification become mandatory in the UK |
| September 2007 | Additional management support provided to strengthen infection control team and appointment of new infection control doctor |
| October 2007 | New community acquired pneumonia (CAP) policy - levofloxacin and benzylpenicillin for severe CAP instead of ceftriaxone |
| November 2007 | Antimicrobial point prevalence audits introduced to identify inappropriate antimicrobial use |
| December 2007 | Actichlor Plus combined detergent and Sodium hypochlorite solution adopted across the Trust for environmental cleaning by nursing and domestic staff |
| February 2008 | Mattress audit resulted in 75% of mattresses across the Trust being replaced. Systematic mattress audit process implemented |
| April 2008 | Antimicrobial point prevalence audits identify inappropriate use of levofloxacin. Decision made to restrict levofloxacin use on all wards except A&E and AMAU |
| July 2008 | Medical cohort ward opened to accommodate patients with primary diagnosis of CDI |
| August 2008 | New antimicrobial formulary launched focusing on reducing cephalosporin use |
| November 2008 | Purpose-built, 13 bedded isolation ward opened to accommodate symptomatic patients with diarrhoea and vomiting (replaces CDI cohort ward) |
| December 2009 | Introduction of dry mist hydrogen peroxide vapour (HPV) fogging (rooms evacuated only) |
| December 2009 | Department of Health report: “*Clostridium difficile infection: How to deal with the problem”* is adopted by the Trust |
| December 2009 | Wards required to isolate patients within 4 hours of confirmation of result or earlier if CDI suspected |
| May 2010 | Deep cleaning program extensively implemented.  Establishment of a decant ward to allow a rolling programme of simultaneous deep cleaning of entire wards rather than individual bays or rooms |

**Supplementary Figure 1.** ***Clostridium difficile* Testing at the Royal Liverpool and Broadgreen University Hospitals NHS Trust (RLBUHT) between July 2008 and May 2010.**

**Supplementary Figure 2. Un-rooted neighbor-joining phylogeny of *C. difficile* 027/ST1 isolates.** Nodes are colored according to isolate groupings (lineage I, II, III and IV). Numbers on key branches represent positions of SNPs based on the reference genome of *C. difficile* R20291. The colored lines to the right of each node indicate type of the corresponding SNP genotypes.

**Supplementary Figure 3. Classification of types of transmission events [**[**2**](#_ENREF_2)**]**

**Supplementary Figure 4. Venn diagrams demonstrating overlap of transmission events detected with a MLST genotype (yellow) and SNP genotypes (blue).** In contrast to the transmission events detected with a MLST genotyping method (without the phylogeny of 027/ST1 isolates), SNP genotyping allowed us to recognize 35 overestimated transmission events and 16 missed transmission events by MLST genotyping. These overestimated events or missed events by MLST genotyping occurred within specific ward as well as between specific wards.

**Supplementary Figure 5.** **Movement data for all identified potential donors that are classified into three categories (triple, double and single) based on infecting number of patients**. Numbers of donors related to these three categories are shown in parentheses. P-values were calculated using unpaired two-tailed t-test. Only significant P-values at significance P < 0.05 are shown. Error bars represent mean ± S.D.

**Supplementary Figure 6.** **Treatment data during stay in hospital for all identified potential donors that are classified into three categories (triple, double and single) based on infecting number of patients**. Numbers of available donors related to these three categories are shown in parentheses. P-values were calculated using unpaired two-tailed t-test (P <0.05). Error bars represent mean ± S.D. None of the treatment data was significant at P <0.05.

**Reference:**

1. He M, Miyajima F, Roberts P, et al. Emergence and global spread of epidemic healthcare-associated Clostridium difficile. Nature genetics 2013; 45(1): 109-13.

2. Walker AS, Eyre DW, Wyllie DH, et al. Characterisation of Clostridium difficile hospital ward-based transmission using extensive epidemiological data and molecular typing. PLoS medicine 2012; 9(2): e1001172.
